# Optimization Based on the Surface Plasmon Optical Properties of Adjustable Metal Nano-Microcavity System for Biosensing

**DOI:** 10.3389/fchem.2021.762638

**Published:** 2021-10-15

**Authors:** Jin Zhu, Yiye Yang, Yanping Yin, Huining Yuan

**Affiliations:** ^1^ School of Electronic Information, Jiangsu University of Science and Technology, Zhengjiang, China; ^2^ School of Electronic Science and Technology, Xiamen University, Xiamen, China

**Keywords:** silver nanorod, surface plasmon resonance, optical properties, metal film coupling, biosensor

## Abstract

This paper mainly studies the plasma optical properties of the silver nanorod and gold film system with gap structure. During the experiment, the finite element analysis method and COMSOL Multiphysics are used for modeling and simulation. The study changes the thickness of the PE spacer layer between the silver nanorod and the gold film, the conditions of the incident light and the surrounding environment medium. Due to the anisotropic characteristics of silver nanorod, the microcavity system is extremely sensitive to the changes of internal and external conditions, and the system exhibits strong performance along the long axis of the nanorod. By analyzing the extinction spectrum of the nanoparticle and the electric field section diagrams at resonance peak, it is found that the plasma optical properties of the system greatly depend on the gap distance, and the surrounding electric field of the silver nanorod is confined in the gap. Both ends of the nanorod and the gap are distributed with high concentrations of hot spots, which reflects the strong hybridization of multiple resonance modes. Under certain excitation conditions, the plasma hybridization behavior will produce a multi-pole mode, and the surface electric field distribution of the nanorod reflects the spatial directionality. In addition, the system is also highly sensitive to the environmental media, which will cause significant changes in its optical properties. The plasma microcavity system with silver nanorod and gold film studied in this paper can be used to develop high-sensitivity biosensors, which has great value in the field of biomedical detection.

## Introduction

The outbreak of a new type of coronavirus pneumonia in 2020 has made mankind deeply aware of the fragility of life, and also made people see the infinite possibilities of modern medical technology. With the increase in the number of patients and the demand for virus detection, the application of surface plasma technology in biosensing (Chevaliez et al., 2021) and medical detection (An et al., 2021) has become the research hotspots.

The free electrons on the surface of metal nanoparticles can excite the surface plasmon resonance effect under specific external conditions. The formation of metal particles and other materials into a new type of nanostructure with small gap can break through the limitations of the previous optical research field and compress the light field energy to a space of a few nanometers. At this time, a strong local electric field enhancement effect is generated around the nanoparticles (Lee et al., 2016). So as to realize the local control of the electromagnetic field energy at the nanometer level (Chen et al., 2015).

At present, scientific research is committed to combining the surface plasmon resonance with biological and medical technologies. The research directions that have attracted international attention include the detection of small biological molecules, the identification of diseases and the drug researches. Tanvir and his team studied the surface plasmon resonance biosensor based on hexagonal lattice dual-core photonic crystal fiber (Ahmed et al., 2019). Besides, Malsagova and others have developed a nanowire biosensor for the detection of hepatitis C virus (Malsagova et al., 2020). The team of Islam Mohammad Rakibul explored a highly sensitive gold-plated PCF biosensor based on surface plasmon resonance (Islam et al., 2021), which is helpful to the improvement of medical diagnostic technology. During the past few years, scientists have made significant progress in the research of plasma biosensors for virus detection. Sharma Pushpendra K and others have developed a surface plasmon resonance sensor for detecting Ebola virus (Sharma et al., 2020). More recently, a new coronavirus detection chip based on nano-plasma optical sensing technology has been proposed by Huang and others (Huang et al., 2021), which has made a significant contribution to epidemic prevention and control. Additionally, the surface plasmon resonance technology has made major breakthroughs in biological imaging (Shpacovitch et al., 2015), DNA separation (Liu and Huang, 2013) and detection of small molecules (Prabowo et al., 2020). It also makes contributions to the production of hyperthermia reagents for cancer (Rozanova and Zhang, 2009) and research in the field of molecular biology.

Based on the existing research results, it is found that surface plasmon technology can be well applied to Raman scattering enhancement and the production of high-sensitivity biosensors (Masterson et al., 2021). In recent years, some new types of nano-metal structures have appeared. It is expected that metal nanostructures with stronger resonance effects, such as porous nanometers (Fan et al., 2020), 2D materials (Zhao et al., 2021) and spiral structures (Wang et al., 2020), can be coupled to thin film substrates, which may be able to obtain unexpected effects (Song et al., 2018). Because the structure of silver nanorod is anisotropic, they are very sensitive to changes in the internal structure of the system and external conditions. Silver nanolabels provide a convenient route for the construction of ultrasensitive electrochemical immunosensor. In the visible range, the imaginary part of the dielectric function of silver is very small, which is the plasmon material with the smallest loss in the visible region. Therefore, compared with other metal nanocrystals silver nanocrystals and exhibit weaker plasma damping and greater light scattering, so they are easier to resonate. Using gold nanoparticles or graphene as deposition matrix, silver deposition has been used for the detection of biomarker antigen, DNA and RNA, as well as bacteria (Lin et al., 2014; Liu et al., 2021). In this work, the silver nanorods have become the preferred experimental objects for studying the characteristics of surface plasmon resonance.

So far, however, there has been little discussion about the gap structure of nanoparticle systems or the complex surface plasmon optical properties of these systems in the field of medical detection. In addition, little is known about the surface plasmon resonance phenomenon of multi-mode coupling. Li et al. demonstrated that the polarization dependent scattering radiation of the film-coupled nanosphere dimer can be used to optically distinguish from monomers and concurrently determine the spatial orientation of the dimer with significantly improved accuracy at the single-particle level. (Li et al., 2016). Sugimoto et al. found that plasmonic nanoparticle on mirror antennas with sub-10 nm gaps have shown the great potential in nanophotonic applications because they offer tightly confined electric field in the gap and resultant large Purcell factors (Sugimoto et al., 2018). The near-field and far-field optical responses of nanoparticle-on-film systems have been studied recently. The associated near-field enhancement in the gap between the particle and the film strongly depends on the film thickness (Armstrong et al., 2019). Wang et al. proposed a surface plasmon resonance fiber biosensor using multi-layer gold nanoparticles/Au film coupling to enhance sensitivity (Wang X. et al., 2021).

**FIGURE 1:**
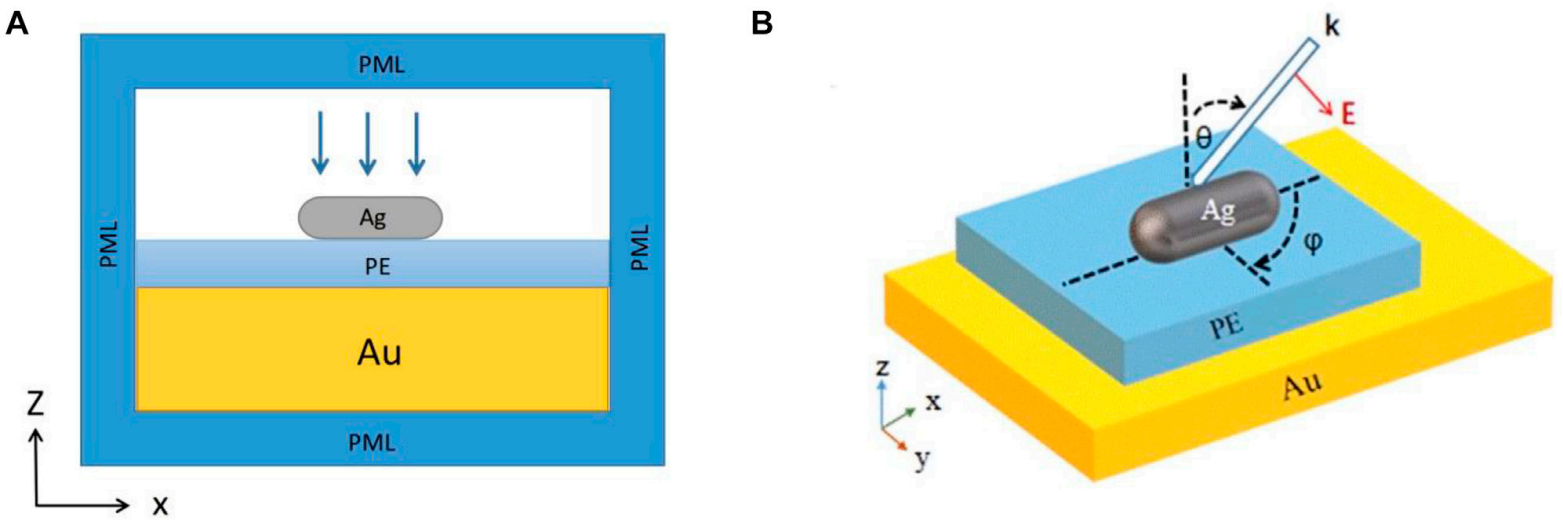
**(A)** The X-Z section of silver nanorod and gold film plasma microcavity model. **(B)** Three-dimensional view of the silver nanorod and gold film plasma microcavity model.

The wavelength of the surface plasmon resonance peak is closely related to the density of electrons, the effective mass of electrons and the distribution of charge. The excitation effect of the surface plasmon will be affected by the shape and structure of metal nanoparticles and the surrounding environment. In this paper, a microcavity structure with the silver nanorod and gold film is studied. The theoretical model is shown in Figure 1A. The high amplitude characteristics of its longitudinal dipole and excellent colloidal stability and biocompatibility help to optimize and improve the performance of the sensor. This paper mainly studies the optical properties of the gap structure of the silver nanorod and gold film system, by changing the gap distance inside the system, the external conditions and the surrounding environment medium. By using the combination of biomolecules to obtain amplified signals, this research will provide a theoretical basis for the development of ultra-sensitive biosensors and nano-optical technologies in real-time diagnostics (Ho et al., 2005). Additionally, it is hoped that the system can be applied in the research of fluorescence sensing (Uppa et al., 2018), surface-enhanced Raman scattering (SERS) (Mueller et al., 2021)and plasmon ruler (Li et al., 2018).

## Numerical Simulation

The research model is based on the Mie scattering theory proposed by Gustav Mie (Mie, 1908). Mie theory is used to calculate the scattering, absorption and extinction characteristics of a single medium spherical particle in a homogeneous medium under the irradiation of a monochromatic parallel beam. In the study, COMSOL Multiphysics 5.5 is used to simulate the extinction cross section and electric field distribution of the silver nanorod and gold film model. The numerical analysis method adopts the finite element method FEM. The basic principle of this method is to divide the research object into multiple subunits, and each small unit domain contains a simple function of finite parameters. After the numerical equations of each small unit are solved, the set of the data is the approximate solution of the research object.

Since the existence of the gold film breaks the uniformity of the background field of the nanorod, the background field method used in the simulation is divided into two steps. The first step is to use the Fresnel equation to calculate the three-layer media structure, including the environmental dielectric layer (na = 1), the gold film (Au) (Johnson and Christy, 1972) and the intervening polyelectrolyte layer (PE, nb = 1.58) (Mock et al., 2008). The polyelectrolyte layer is between the gold film and the nanorod. The full-field solution without nanoparticle scatterers is obtained by simulation. In the second step, the silver nanorod is set to the active state, and the field without scatters obtained in the first step is used as the background field where the silver nanorod-gold film model is located. In this way, Maxwell’s equations can be solved successively to calculate the silver’s extinction cross-section data and surface electric field distribution of the nanorod.

As shown in Figure 1B, in all simulations, the silver nanorod (Ag) (Johnson and Christy, 1972)consists of a cylinder and two hemispheres. The height of the cylinder is 75 nm, and the diameter of the hemisphere is 30 nm. The air domain is 400 nm in length, 400 nm in width and 200 nm in height. The spacer layer (PE) is 400 nm in length and 400 nm in width, while the thickness is adjustable. The gold film (Au) is 400 nm in length and 400 nm in width, and the height is 45 nm. In order to reduce the interference of reflection and improve the convergence speed of calculation, the outermost periphery of the physical domain is wrapped with a layer of PML domain, and its thickness is set to 80 nm. The initial variables in the simulations are defined as follows. The spacer layer between the nanorod and the gold film is named PE, and its thickness is 12 nm. In Figure 1B, the angle between the projection of the incident wave vector in the X-Y plane and the *x*-axis is defined as the azimuth angle *φ*. The modeling parameter name is phi, and the initial value is 0°. The angle between the normal of the substrate and the incident wave vector in Figure 1B is defined as the incident angle *θ*, and its modeling parameter name is theta with the initial value 0°. The nanorod is in the air.

Since the study uses the controlled variable method for experiments, only one parameter value is changed each time for simulation. The variable settings are as follows. First, change the thickness of the polyethylene spacer layer. The thickness should increase linearly with the number of deposited double layers (approximately 4.1 nm) (J et al., 2008), and the research range is from 4 to 28 nm with a step length of 4 nm. Second, change the value of the azimuth angle *φ*. The research values are 0°, 30°, 60°, 85° (approximately instead of 90°). Third, change the value of the incident angle *θ*. The study range is from 0° to 85° (approximately instead of 90°) with a step length of 10°. Fourth, change the environmental medium where the nanorod is located. The experiment selected air, water, glass, and polyether ether ketone (PEEK) for simulation.

## Results and Discussion

### Research on the Thickness of PE

**FIGURE 2:**
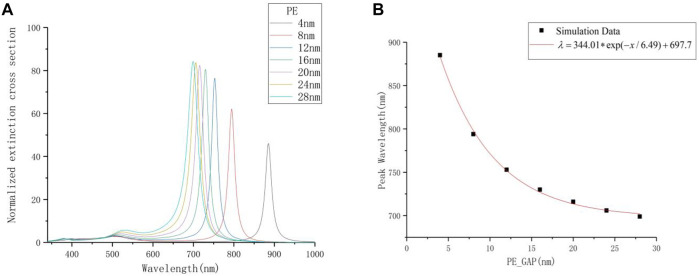
**(A)** The extinction spectrum with different thickness of PE. **(B)** The relationship between the thickness of PE and the resonance peak.

In this experiment, the thickness of PE is changed. The research range is from 4 to 28 nm, and each step length is 4 nm. The incident angle and azimuth angle are both set to 0° here in order to simulate the vertical incidence condition, and the environment medium is air. To facilitate the analysis of the experimental data, the extinction spectrum is normalized. In Figure 2A, there are two absorption bands. The longitudinal resonance peak is located in the range of 650–900 nm, while the transverse resonance peak is located near 520 nm. They respectively reflect the effect of the free electrons on the surface of the silver nanorod moving along the long axis and the short axis of the nanorod. According to this, the long-wave resonant peak exhibits strong anisotropic polarization characteristics related to the long-axis resonance. This is because the collective resonance of free electrons along the long axis enhances the probability of inter-band radiation transition, causing the intensity of its oscillator become relatively large, while the short-wavelength resonance does not change obviously. The electric field intensity around the nanorod at the longitudinal resonance peak changes significantly, so the resonance peak plays a major role in the optical properties of the nanorod.

With this structure, the hybridization between the localized surface plasmon resonance of metal nanoparticle and the propagating surface plasmon of the metal substrate is extremely sensitive to the gap distance. As the thickness of PE decreases, the resonance peak of the longitudinal mode gradually redshifts, and the amplitude gradually decreases. When the interval is uniformly reduced, the redshift speed of the formant and the rate of decrease in amplitude both accelerate significantly. When the thickness of PE is reduced to 4 nm, its resonance peak is already in the infrared band, but the extinction intensity is relatively low. Preliminary speculation is that the increase of the phase retardation between the silver nanorod and the gold film makes the resonant peak redshift, and at the same time the extinction cross section is reduced.

The relationship of different thickness of PE (PE_GAP) and their corresponding resonance peaks (Peak Wavelength) is shown in Figure 2B. As the thickness of PE decreases, the corresponding resonance peak wavelength increases exponentially. This is because the intensity of the anti-parallel mirror dipole induced on the substrate is extremely sensitive to changes in small distance. Here, a single exponential decay function is used to fit the data set, and the relationship is:

λ=344.01∗exp(−x/6.49)+697.7 (1)

In order to better reveal the optical and physical properties of the plasma mode related to the scattering maximum in the simulated spectrum, the electric field distribution map of the surface of the nanoparticle at the resonant peak at different gap distances is calculated here. This article mainly observes the Ex, Ey, Ez and |E| components of each section. Both the Z-X and Z-Y sections pass through the center of the silver nanorod. The X-Y section is the upper surface layer of the gold film.

In Table 1A, the Ex and Ez components gradually radiate to the space outside the nanorod as the gap increases. The electric field component at both ends of the nanorod and in the gap shows a significantly weakening trend. Due to the anisotropy of the nanorod, different charge distributions are produced on the surface of the nanoparticle. Ex and Ez components both show that the quadrupole mode appears on the surface of the nanorod. At this time, there is almost no Ey component in the Z-X section. The area with high intensity brightness in the picture of |E| component is called “hot spot”. There are hot spots with higher concentration of electric field at the gap and both ends of the nanorod. The reason for this electromagnetic focusing effect is that the existence of the metal film substrate breaks the scattering characteristics of the rod-shaped silver nanoparticle, and there is a close connection between the silver nanoparticle and gold film. In Table 1B, the electric field component of the Z-Y section changes very little with the change of the PE’s thickness. Based on this, it is inferred that the excitation of the transverse mode hardly depends on the change of the gap distance of the system. In Table 1C, the upper surface of the gold film also shows a quadrupole mode, which is consistent with the pictures of Ex and Ez components shown in Table 1A.

**TABLE 1:** The electric field section at the resonance peak at different PE interval thicknesses.

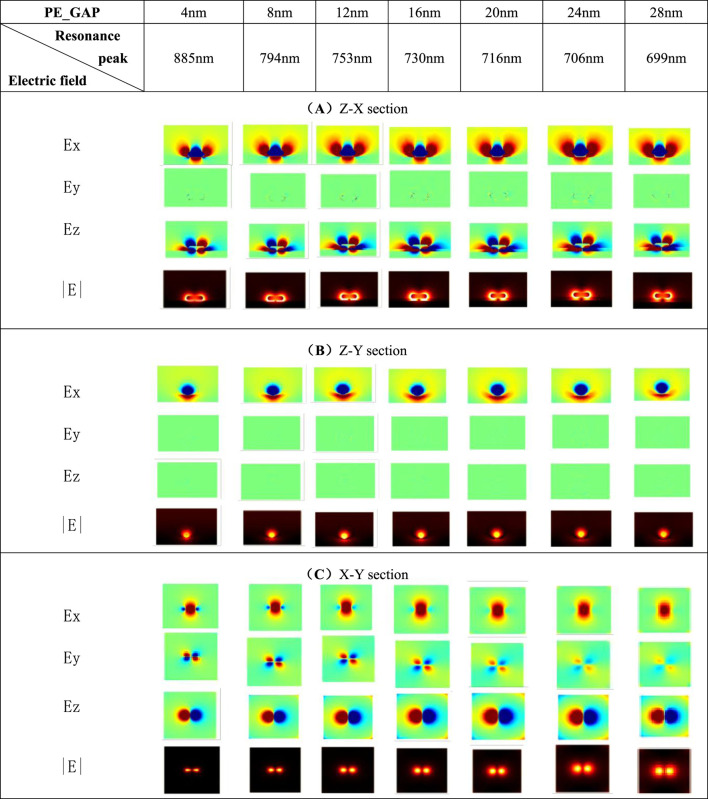

The silver nanorod is highly sensitive to changes in the gap distance. When the gap is small, the electric field is mostly confined to the two ends of the nanorod and the gap. These areas show enhanced local electric field effects. And there is a strong dipole resonance along the long axis of the nanorod. When the gap is large, the electric field has some expansion at both ends of the nanoparticle, and the concentration of hot spots in the system will decrease as the gap increases. The mode of particle-film coupling structure can be used as a plasmonic nanometer measurement tool, which is convenient for further research on plasma-mediated optical sensing and enhancement. And it can highly improve the sensitivity of nano biosensors.

### Research on the Azimuth Angle

This experiment changes the azimuth angle *φ*, using the values of 0°, 30°, 60°, 85° (approximately instead of 90°). The incident angle *θ* here is set to 0°, and the environment medium is air. In order to explore the sensitivity of different gap thicknesses to azimuth angle changes, the PE gap thickness is changed to 4, 8, and 12 nm for simulation. The result well reflects the difference in plasmon resonance effect between the large-gap structure and the small-gap structure.

**FIGURE 3:**
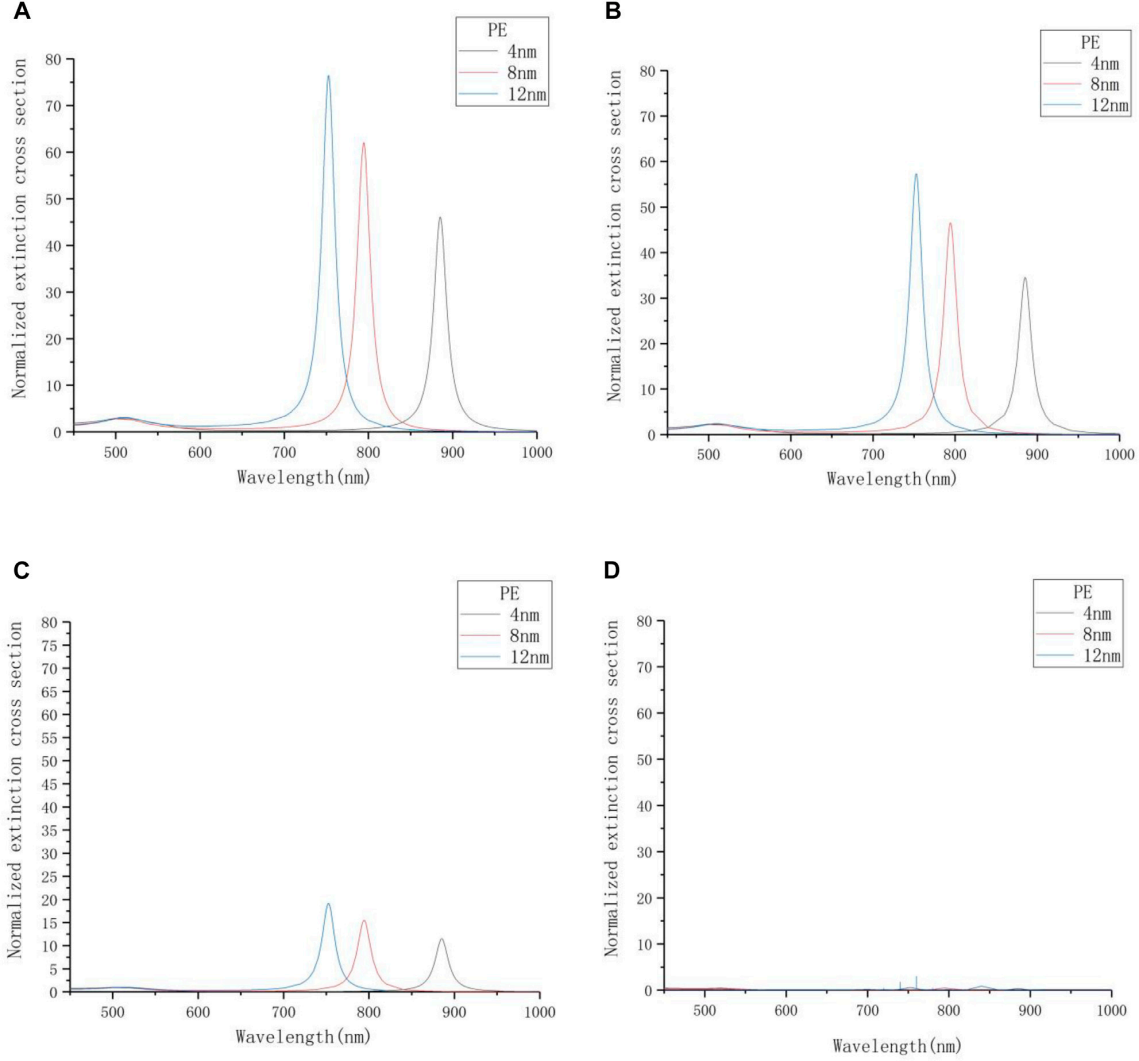
The extinction spectrum with different azimuth angles. **(A)** *φ* = 0°. **(B)** *φ* = 30°. **(C)** *φ* = 60°. **(D)** *φ* = 85°.

Figure 3 shows the influence of the azimuth angle *φ* on the extinction characteristics of the system with different PE thicknesses. In Figures 3A–C, as the angle gradually increases, the extinction intensity of the nanoparticles in each gap structure gradually decreases. Figure 3D shows that the extinction intensity is close to 0 when the angle increases to 85°.

**FIGURE 4:**
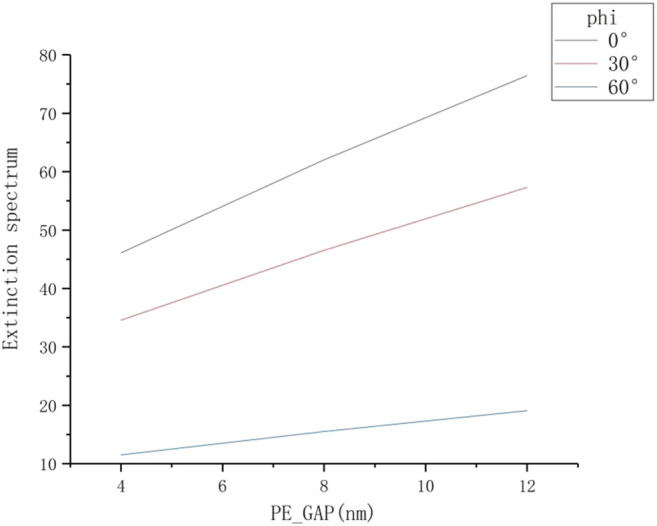
The extinction spectrum with different thickness of PE under different azimuth angle conditions.

Statistics of the data obtained in Figure 3 are shown in Table 2. It is observed that if the thickness of PE is fixed, no matter how the angle *φ* is changed, the position of the plasmon resonance peak of the nanorod will not change. At the same time, as the azimuth angle *φ* increases, the extinction intensity of the nanorod resonance peak will decrease. The data related to thickness of PE, extinction intensity and azimuth angle in Table 2 are plotted as Figure 4, and three straight lines with different slopes are obtained by fitting. As the thickness of PE increases, the extinction intensity of the nanorod shows a linear and monotonous growth trend. When *φ* = 0°, the slope of the fitted line is the largest, indicating that the unit increase of thickness has a strong influence on the extinction intensity of the resonance peak, so the optical sensitivity of nanoparticles under this incident condition is relatively higher.

**TABLE 2:** Data of extinction intensity at the longitudinal resonance peak under different PE thicknesses.

PE (peak wavelength) extinction spectrum *φ*	4 nm (885 nm)	8 nm (794 nm)	12 nm (753 nm)
0°	46.08	62.02	76.43
30°	34.56	46.52	57.31
60°	11.52	15.51	19.10

The structure of PE = 12 nm is selected in order to facilitate the observation of the surface electric field distribution around the nanorod at different azimuth angles, and the electric field sections at the resonance peak of 753 nm are drawn in Table 3. According to the |E| component in Table 3, when *φ* = 0°, the charge concentration on the surface of the nanorod is relatively large. Two strong hot spots are generated in the gap, and the local electric field strength is greatly enhanced. With the gradual increase of *φ*, the surface charge concentration of nanoparticle decreases, and the electric field strength gradually weakens. When *φ* = 85°, the weakening effect of each electric field component around the nanorod is significant. Based on this, as the azimuth angle value increases, the local electric field intensity around the silver nanorod shows a decreasing trend. The surface electric field distribution characteristics are consistent with the extinction spectrum, so under the premise that other conditions are not changed, the system’s plasmon resonance excitation effect is the best when the incident condition is vertical (*φ* and *θ* both are 0°).

**TABLE 3:** Cross-section diagrams of electric field under different azimuth angles.

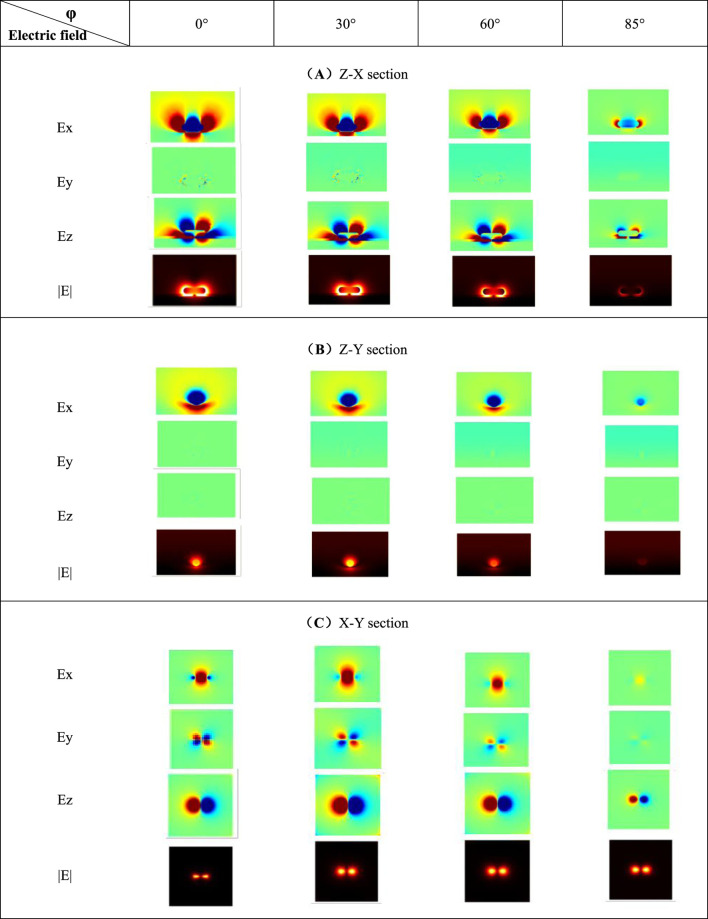

### Research on the Incident Angle

**FIGURE 5:**
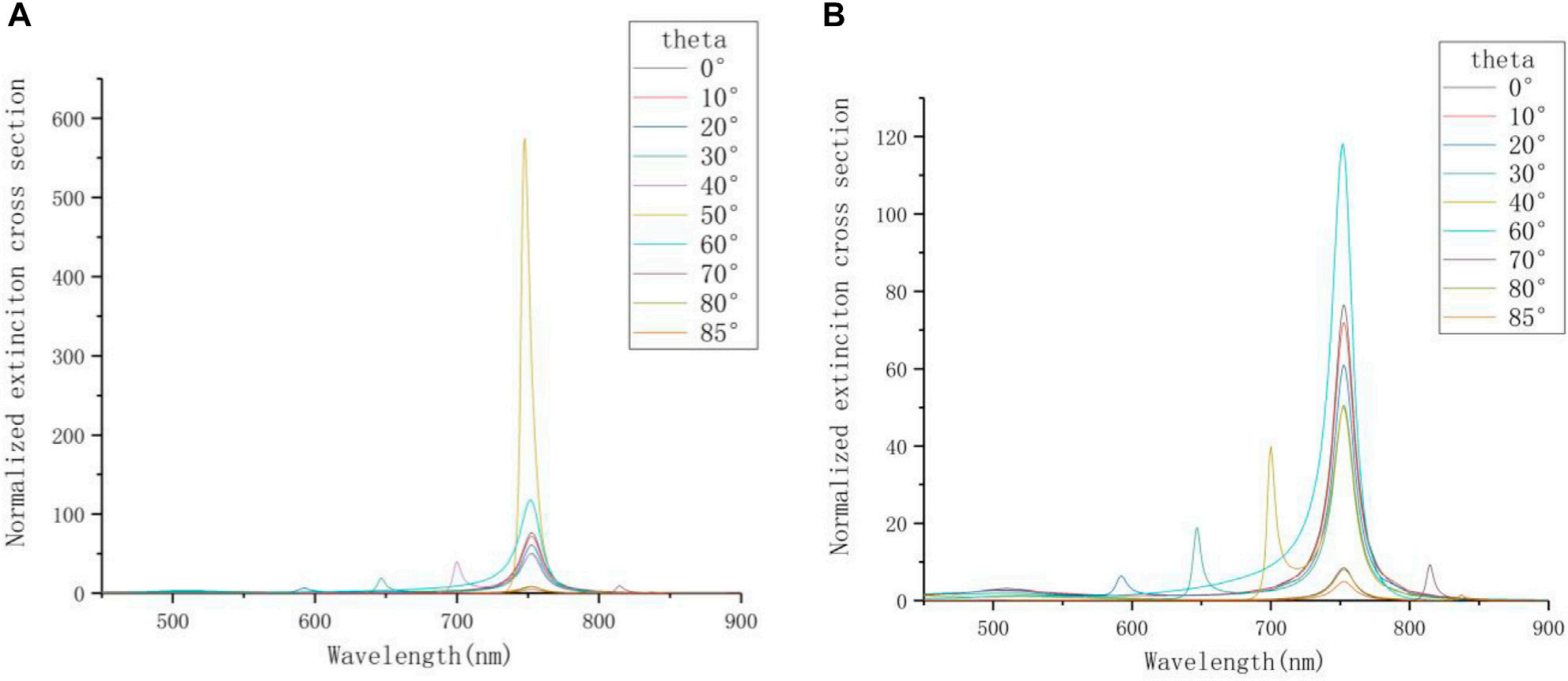
**(A)** The extinction spectrum at different incident angles. **(B)** The extinction cross sections at different incident angles except *θ* = 50°.

This experiment changes the incident angle *θ* for simulation. The research range of incident angle is from 0° to 85° (approximately instead of 90°), and the step length is 10°. The azimuth angle *φ* is set to 0°. The PE thickness is 12 nm here, and the environment medium is air. The main purpose is to analyze the influence of different incident angles on the plasma characteristics of the system. The extinction spectrum is shown in Figure 5A. The extinction intensity of the resonance peak is greatly enhanced when *θ* = 50°, which is 5–10 times that of other incident conditions. It is preliminarily speculated that the near-field characteristic of the plasma mode is based on the mixing of multi-pole modes, which forms a strong asymmetric dipole moment on the surface of the film and enhances the extinction cross section, indicating that the radiation mode generated in the system strongly depends on the incident polarization.

In order to see the details of the extinction spectrum of other incident conditions more clearly, the curve of *θ* = 50° is deleted in Figure 5B. When some specific values are taken, the extinction spectrum will produce multi-peak phenomenon. It can be speculated that the highly restricted gap distance between the gold film and the nanorod simultaneously excites surface plasmon resonance and localized surface plasmon resonance (Chen et al., 2015). The two resonance effects are mixed and can excite very complex plasmas under different incident angles. As a result, the surface plasmon resonance in the vertical mode occurs. The resonance peak between 550 and 700 nm is the vertical mode excited by changing the incident angle. This is consistent with the researches on theoretical conversion optics (Sugimoto et al., 2018).

In Table 4, it is found that when *θ* = 50°, the |E| component shows there are strong hot spots near the nanorod, and the local electric field enhancement effect on the surface is significant. On the one hand, the Ez component of the Z-X section when *θ* = 50° shows that the blue part radiates into the air from the surface of the metal film and it exhibits exponential attenuation. It is speculated that the oblique incident angle can excite the surface plasmon polaritons (SPP). At this time, the coupling effect of LSPR and SPP greatly enhances the electric field intensity on the surface of the nanorods (Zouheir et al., 2016). On the other hand, the spectrum at *θ* = 50° shows that there is only one resonant peak in the longitudinal mode, and its position is shifted by 5 nm compared with other spectra with the hybrid multimodal phenomenon. Compared with the case of *θ* = 20°, the weak resonance peak of *θ* = 30° has a red shift on the extinction spectrum. Therefore, it is speculated that the position of the resonance peak generated by the hybridization at *θ* = 50° is very close to the resonance peak of the longitudinal mode (at 753 nm). In the extinction spectrum, the two resonance peaks are merged into one, so only one peak with high intensity can be observed. When *θ* is set to 40° and 70°, the electric field distribution of the nanorod in hybrid mode is different. In this type of mixed mode, the electric field distribution around the nanorod shows complementary optical characteristics at the two resonance peaks. Observing the Ex, Ey, and Ez components, it is found that the polarities of the electric dipoles distributed on the surfaces of the two resonance peaks are opposite, which is not present on the extinction spectrum. The Ex and Ez components in Table 4A show that after changing the *θ*, the electric field distribution at both ends of the nanorod is asymmetric and has spatial directionality. If the components corresponding to the electric field sections under the two resonance peaks are respectively superimposed, it is found that the electric field component diagram after the two fusions is very similar to the electric field component diagram without multi-peak phenomenon when *θ* takes 0°. Based on this, when the incident light is oblique, specific angles can make the longitudinal axis mode resonant peak of the nanorod hybridize, which reflects the multimodal phenomenon in Figure 5B. Besides, Table 4B shows that when the *θ* is set to 40°, 50° and 70°, the Ey and Ez components appear on the Z-Y section. And the |E| component has also been enhanced.

**TABLE 4:** Electric field cross-section at different incident angles.

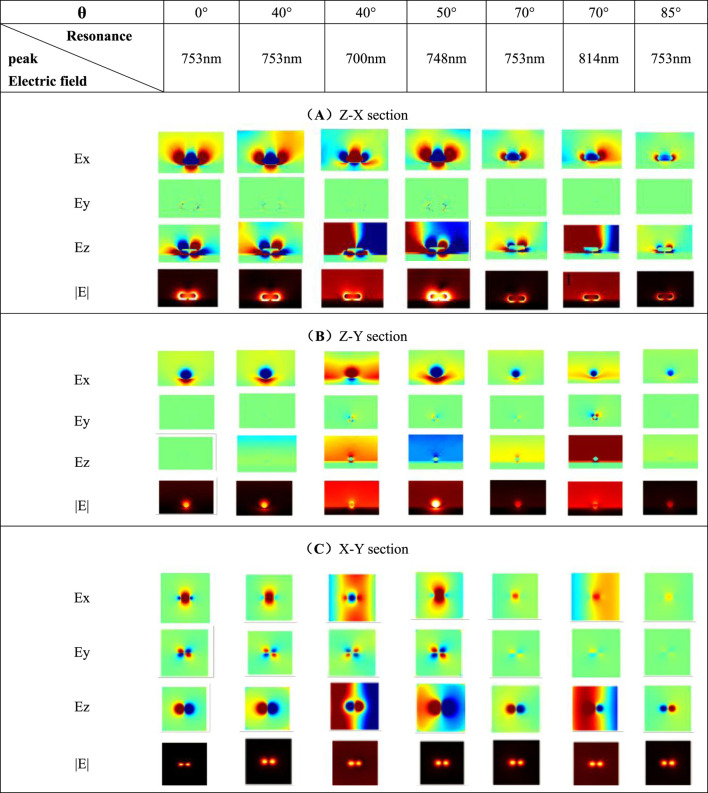

The complex plasmon resonance mode has mixed multipolar components. Since the radiation damping is proportional to the square of the charge acceleration in the system, the total radiation damping in the interacting plasma structure will exhibit interference effects. When the structure of the particle-film system is relatively complex with different incident conditions, the interaction of multiple modes will produce a hybrid effect, which makes the dipole moment larger and enhance the surface local electric field. Therefore, it is inferred that changing the degree of incident angle can affect the excitation of the multimodal mixed mode. By artificially adjusting the incident conditions and modulating the proportions of different modes, a specific surface plasmon resonance mode is obtained. And a high-sensitivity detection technology can be developed to capture information in a specific wavelength band in order to improve the sensor’s signal detection ability.

### Research on the Refractive Index of the Environment Medium

**FIGURE 6:**
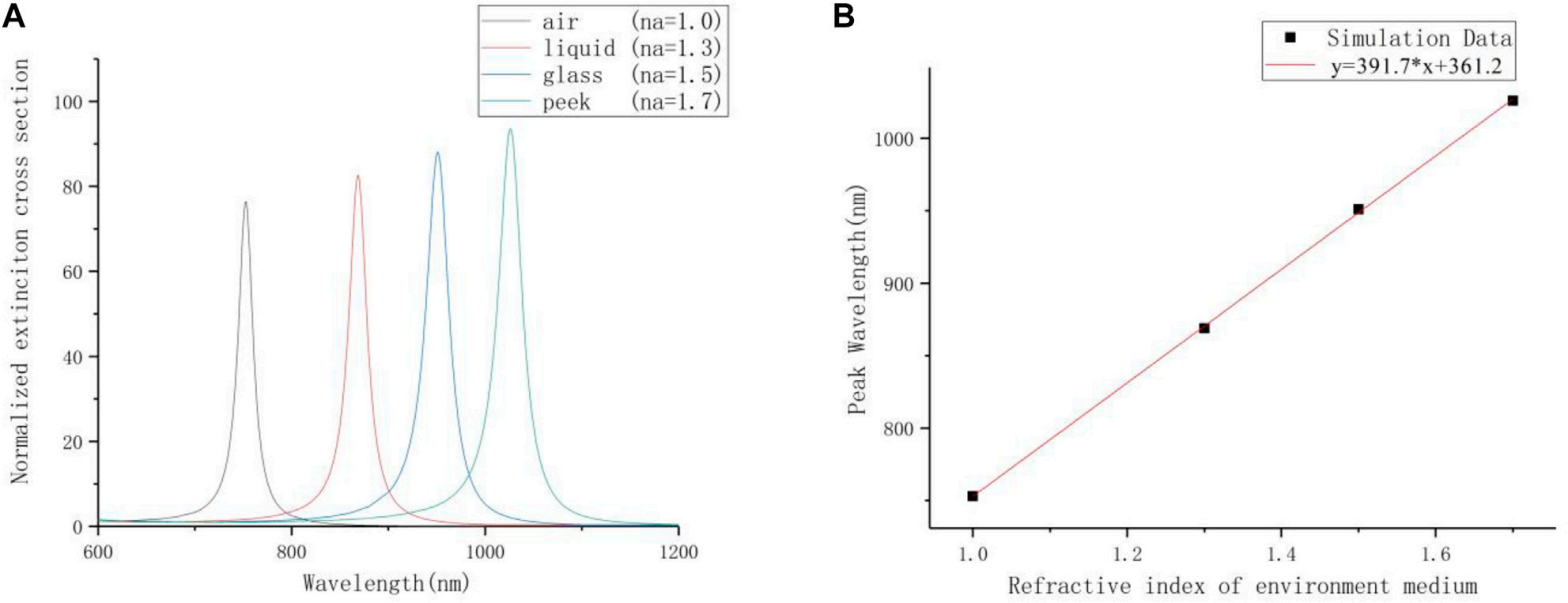
**(A)** The extinction spectrum with different environmental media. **(B)** The relationship between the refractive index of the environmental media and the resonance peak.

This experiment changes the environment medium. The research selects air (na = 1.0), water (na = 1.3), glass (na = 1.5) and polyether ether ketone (na = 1.7, simulated blood environment). The incident angle and the azimuth angle are both set to 0°, and the PE thickness is 12 nm. The extinction spectrum is shown in Figure 6A. As the refractive index of the environment medium increases, the resonance peak of the longitudinal mode redshifts, and the intensity gradually increases. When the system is in the environment of glass and polyether ether ketone, the resonance peak is already in the infrared band. Based on the results, it can be inferred that when the thickness of PE is adjusted to a smaller distance (<10 nm), the resonance peak will continue to redshift.

According to the extinction spectrum, the relationship between the resonance peak and the environmental medium is fitted in Figure 6B. The shift of the longitudinal mode resonance peak and the change in the refractive index of the environmental medium present an approximately linear relationship. The fitting expression is shown in the following equation:

This optical characteristic can be used to make a nano sensor. The environmental-refractive-index sensitivity of the above sensor is 391.7 nm/RIU. If further research is carried out, a highly sensitive infrared sensor can be obtained, which is used to sense small changes in environmental parameters, such as temperature, PH value and cell metabolism rate.

## Conclusion

The excitation of metal nanostructure plasmon resonance is affected by factors such as the size of nanoparticle, the external incentive conditions, and the surrounding environment medium. In the system of the silver nanorod and gold film with the coupling gap structure studied in this paper, the surface electric field is greatly confined in the gap, and the complex multi-pole mode can be excited under different external incident conditions. That reflects the strong coupling effect of multiple resonance modes.

Based on the analysis of experimental results, the nanorod-film system with the gap structure has a strong surface plasmon resonance effect and a local electric field enhancement in the gap when the gap is small (PE = 4 nm). The characteristics can be used to enhance surface Raman scattering and reduce ohmic losses. At the same time, the exponential relationship between the gap thickness and the resonance peak position can be used to make a highly sensitive plasmon ruler, which is convenient for accurate detection of small molecular substances such as DNA. When the incident angle *θ* is set to 0°, the extinction intensity of the nanorod decreases with the increase of the azimuth angle *φ*. And the extinction intensity increases linearly with the increase of the thickness of PE under the same condition of azimuth angle *φ*. So, when the azimuth angle is set to 0°, the surface plasmon resonance excitation effect is the best, and the sensitivity of the system is higher.

If the incident angle *θ* is set to 40° and 70°, the silver nanorod produces a new plasmon resonance mode—the vertical mode. When the incident angle *θ* is 50°, the local electric field on the surface of the nanorod is stronger than other incident conditions. Therefore, by artificially setting the incident angle conditions, different proportions of the surface plasmon resonance and the surface plasmon resonance effect of the multi-pole mixed mode can be modulated. In addition, the sensitivity of the environmental refractive index sensor based on this nanorod-film system is 391.7 nm/RIU.

In conclusion, the silver nanorod and gold film microcavity system studied in this paper has huge research significance in the field of biological nano sensors and the medical detection. Some biological science experiments have proved the value of this work. Takemura confirmed that biosensors based on LSPR are effective in minimizing false-positives and false-negatives that are prevalent in the existing virus detection techniques (Takemura, 2021). In another laboratory, clinical human serum samples were used to verify the practicability of a kind of gold nanoparticle-on-Au film construction biosensor showing great potential value in various clinical applications as a high efficiency, portable and easy to miniaturization point-of-care testing technology (Wang F. et al., 2021). In the future, super-sensitive sensors with better detection performance can be developed to facilitate high-precision detection of DNA, RNA and other biological molecules (Song et al., 2018). Furthermore, it has extremely high application value in manufacturing adjustable plasma sensors, improving research on photothermal conversion, and promoting Raman scattering enhancement.

## Data Availability

The raw data supporting the conclusions of this article will be made available by the authors, without undue reservation.
